# Effects of ethanol combined with ascorbic acid and packaging on the inhibition of browning and microbial growth in fresh‐cut Chinese yam

**DOI:** 10.1002/fsn3.647

**Published:** 2018-04-10

**Authors:** Jia Gao, Yongqing Zhu, Fangyao Luo

**Affiliations:** ^1^ Institute of Agro‐products Processing Science and Technology Sichuan Academy of Agricultural Sciences Chengdu China; ^2^ Ministry of Agriculture Key Laboratory of Biology and Genetic Improvement of Horticultural Crops (Southwest Region) Chengdu China

**Keywords:** ethanol, fresh‐cut Chinese yam, modified atmosphere packaging (MAP), vacuum packaging

## Abstract

The objective of this study was to investigate the effects of aqueous solutions of ethanol (25%, v/v) and ascorbic acid (AA, 1%, m/v) alone and in combination, along with modified atmosphere packaging (MAP) or vacuum packaging (VP), on the physicochemical properties and microbial quality of fresh‐cut Chinese yam slices during 4°C storage. The data showed that ethanol (25%, v/v) combined with AA (1%, m/v) and MAP treatment resulted in the lowest changes for headspace gas composition, color, electrical conductivity, overall visual quality, aerobic plate count population, and mold and yeast population in fresh‐cut Chinese yam over 21 days of 4°C cold storage, which was more effective at both inhibiting microbial growth and delaying browning than ethanol or AA alone or a commonly used sanitizer, sodium hypochlorite. The experiment on packaging demonstrated that samples treated with VP and 25% ethanol with 1% (m/v) AA dip can only preserve the sensory qualities of fresh‐cut Chinese yam slices up to 7 days at 4°C, but 25% (v/v) ethanol in conjunction with 1% (m/v) AA packed with MAP had antibrowning potential and maintained the quality of fresh‐cut Chinese yam slices up to 14 days when stored at 4°C.

## 1 INTRODUCTION

The Chinese yam (shan yao; *Dioscorea opposite* Thunb.) is a popular root vegetable and commonly used like dietary supplement, as well as an herbal medicine in Asia, which contains a variety of phytochemicals, including sapogenins, saponins, starch, mucopolysaccharides, protein, amino acids, and others (Lin & Yang, 2008). Intake of Chinese yam may be beneficial to improve the function of the spleen, stomach, and lung (Huang, Cheng, Deng, Chou, & Jan, 2012). Additionally, Chinese yam has many biological activities, including antidiarrhea, anti‐inflammation, antioxidation, hypocholesterolemia, hypoglycemia, and immunomodulation (Bhandari & Kawabata, 2004; Chen, Wang, Chang, & Wang, 2003; Chiu et al., 2013; Lin, Lu, Liou, & Liou, 2006). In recent years, a great increase in the consumption of fresh‐cut fruit and vegetables, including fresh‐cut Chinese yam, has been observed (Luo, Wang, Jiang, & Xu, 2015). However, processing of fresh produce, such as peeling, cutting, and chopping, could cause spoilage of the vegetable tissues and increase respiration rate, oxidation by phenol metabolism associated polyphenol oxidase (PPO) and peroxidase (POD) enzymes, or microbial growth in the injured tissues (Chen et al., 2017; Luo et al., 2015; Putnik et al., 2017). Especially for Chinese yam, browning and spoilage are both detrimental changes that affect appearance and quality (Krishnan, Padmaja, Moorthy, Suja, & Sajeev, 2010; Luo et al., 2015; Teoh, Lasekan, Adzahan, & Hashim, 2016). Thus, improving the quality and shelf life of fresh‐cut Chinese yam will reduce costs associated with losses and increase demand for this commodity.

Many measures have been used to inhibit enzymatic browning of yam during processing, such as soaking in chemicals (Krishnan et al., 2010), UV‐C exposure (Teoh et al., 2016), electrolyzed water (Lee, Park, Jeong, Kim, & Chinnan, 2007), blanching (Chen et al., 2017), and nano‐CaCO_3_‐LDPE packaging (Luo et al., 2015). However, it has been very difficult up to the present to preserve these products with commercial acceptability beyond a couple of weeks. Therefore, it is necessary to test and incorporate other strategies to increase the shelf life significantly.

Ethanol vapors and dips have been used to sanitize plant materials to inhibit decay and growth of microbial populations in a variety of fresh‐cut fruits and vegetables, such as apple (Bai, 2004), mango (Plotto, Bai, Narciso, Brecht, & Baldwin, 2006), eggplant (Hu, Jiang, Tian, Liu, & Wang, 2010), sweet cherry (Zhang, Samapundo, Pothakos, Surengil, & Devlieghere, 2013), sunchoke tuber (Wang, Nie, & Cantwell, 2014), and lettuce (Yan, Yang, & Luo, 2015). Additionally, some previous studies found that ethanol at a low concentration (20%‐30%, v/v) combined with other antibrowning agents, such as ascorbic acid, was able to both control browning and maintain microbial growth in fresh‐cut lotus root slices (Gao, Luo, Turner, & Zhu, 2017), apples (Yan, Luo, Zhou, & Ingram, 2017), and sugar cane (Homaida, Yan, & Yang, 2017). However, the use of ethanol to maintain microbiological and sensorial quality in fresh‐cut Chinese yam is unknown. Similar to other emerging technologies, the successful application of ethanol combined with ascorbic acid requires that the suitability of application for different products be experimentally assessed. Therefore, the aim of this study was to evaluate the effects of ethanol (25%, v/v) combined with ascorbic acid (1%, m/v) on color, texture, tissue integrity, sensory attributes, and microbial growth in fresh‐cut Chinese yam slices, and to evaluate its ability, in combination with other strategies (modified atmosphere packaging, vacuum packaging and refrigerated storage), to extend shelf life.

## 2 MATERIALS AND METHODS

### 2.1 Plant material

Fresh Chinese yam (*Dioscorea opposite* Thunb.) harvested in November and stored at normal temperature for almost half a month, which is native to Hebei province, China, was purchased from a local wholesale market (Chengdu, China), transported to the laboratory, and stored at 4°C overnight before processing. Samples were selected for uniformity of size and color and the absence of mechanical damage or diseased material.

### 2.2 Sample preparation

Whole Chinese yams were rinsed with tap water to remove soil and washed with sodium hypochlorite (NaOCl) solution (100 mg L^−1^ free chlorine, pH 6.5), which was prepared using Clorox (active chlorine ≥5.5%, Kelong Chemical Reagent Factory, Chengdu, China), and the pH was adjusted using citric acid solution. Chinese yams were hand peeled in one direction using a manual peeler to remove a minimal amount of surface tissue and then sliced 5 mm thick using a vegetable slicer (TYFM Inc., Guangdong, China).

To investigate the effects of ethanol (E) and ascorbic acid (AA) alone and in combination, along with the modified atmosphere packaging (MAP) or vacuum packaging (VP), also compared to a commonly used sanitizer, sodium hypochlorite (NaOCl), seven treatments were prepared including (1) nonchlorinated water wash + MAP, (2) 25% (v/v) E and 1% (m/v) AA wash + MAP, (3) 25% E wash + MAP, (4) 1% AA wash + MAP, (5) NaOCl (100 mg L^−1^ free chlorine, pH 6.5) wash + MAP, (6) nonchlorinated water wash + VP, and (7) 25% E and 1% AA wash + VP. Every 9 kg of fresh‐cut Chinese yam slices was submerged and manually agitated in 10 L of one of the seven treatment solutions for 2‐min immersion. Then, removed excess moisture using a TW‐980S spin‐drier (TYFM Inc., Guangdong, China) and packed. Each bag had 500 ± 2.0 g immersed samples. The MAP treatments used the sealed polyethylene bags (24 cm × 16 cm, Lianyi plastic packaging Inc., Chengdu, China) with selected film O_2_ and CO_2_ transmission rate of 16398.3 cm^3^ m^−2^ 24 hr 0.1 MPa and 68644.9 cm^3^ m^−2^ 24 hr 0.1 MPa. The permeability of the films was tested by Labthink Inc., Shandong, China, at 23°C. The VP treatments used the polyethylene vacuum bags (24 cm × 16 cm, Lianyi plastic packaging Inc., Chengdu, China). All the treatments were stored in the dark at 4°C for 21 days. Package atmosphere, color, tissue electrolyte leakage, texture, sensory evaluation, and microbial analyses were performed on days 1, 7, 14, and 21.

### 2.3 Quality evaluation

#### 2.3.1 Package headspace gas composition

The fractional pressures of O_2_ and CO_2_ in the packages were ascertained using a gas analyzer (Checkmate II, PBI Dansensor Co., Denmark). Without opening each bag, a gas sample was acquired by inserting the needle of a measuring assembly through a septum adhered to the packaging film.

#### 2.3.2 Color assessment

The surface color of the samples was measured with a colorimeter (CR‐400 Chroma Meter, Konica Minolta Optics. Inc., JP). The instrument was calibrated with a white tile (*Y* = 94.0, *x* = 0.3130 and *y* = 0.3191). Measurements were taken for L*, a*, and b* values at two sites on each of 15 yam slices for each treatment group. Color coordinates a* and b* were converted into hue angles [hue = tan^−1^(b/a)].

#### 2.3.3 Electrolyte leakage analysis

Tissue electrolyte leakage was measured following a modified procedure (Wang, Feng, & Luo, 2004). For each package, a sample of fresh‐cut Chinese yam (10 slices, 50 ± 5 g) was submerged in 200 ml of deionized water for 30 min at 22°C. The electrolyte content of the solution was determined by measuring the electrical conductivity with a conductivity meter (model DDSJ‐308A, INESA Instrument, Inc., Shanghai, China). Total electrolytes of the fresh‐cut yam samples were determined after repeatedly freezing at −20°C for 24 hr and thawing at room temperature. Relative electrical conductivity (REC) was expressed as a ratio of fresh over total electrolytes.

#### 2.3.4 Texture analysis

The texture properties of the samples were assessed using a TA.XT Plus texture analyzer (Stable Micro System Corp., UK) with the following parameters: probe = A/MORS shear blade and test speed = 2 mm s^−1^. The cutting force was defined as the average force needed to puncture the Chinese yam slices to a depth of 1.2 mm to 1.8 mm. Measurements were performed at one site in the middle of each fresh‐cut yam slice. For each treatment, 10 slices were measured for each of four replicates.

#### 2.3.5 Sensory evaluation

A four‐member trained sensory panel conducted the visual evaluation of the fresh‐cut Chinese yam samples stored during 21 day. Panel members all had several years of sensory analysis experience with a wide variety of vegetables and fruits. Overall visual quality was assessed with a 9‐point hedonic scale where 9 = like extremely, 7 = like moderately, 5 = neither like nor dislike, 3 = dislike moderately, and 1 = dislike extremely (Bai, et al. 2004). The samples were coded with a random three‐digit number to mask the treatment identity in order to minimize subjectivity and were compared with freshly prepared samples. Samples were not tasted.

### 2.4 Microbial assays

Each sample consisted of five slices (20 ± 4 g) was blended with 225 ml of sterile physiological saline (8.5 g L^−1^ NaCl) using a sterile stomacher bender (Qingdao Hope Bio‐Technology Co., Ltd, Qingdao, China) for 2 min at high speed. A 100 μl sample of each filtrate and its appropriate dilution was spread on agar plates. The aerobic plate count (APC) was determined by plating samples on plate count agar (Aoboxing Bio‐Technology Co., Ltd, Beijing, China) and incubated at 37°C for 24 hr. Mold and yeast (M&Y) enumeration was performed by plating on potato dextrose agar (Aoboxing Bio‐Technology Co., Ltd, Beijing, China) supplemented with 200 mg L^−1^ chloramphenicol (Solarbio Science & Technology Co., Ltd, Beijing, China) and incubated at 28°C for 48 hr. Microbial colonies were reported as log CFU g^−1^ of tissue.

### 2.5 Statistical analysis

Four replications (four bags) per treatment were evaluated on each evaluation day. Data were analyzed using SPSS 16.0 using a two‐factor (treatment and storage duration) linear model. All data are reported as the mean of four replicates ± standard error (*SE*).

## 3 RESULTS AND DISCUSSION

### 3.1 Assess efficacy of wash treatments

**Figure 1:**
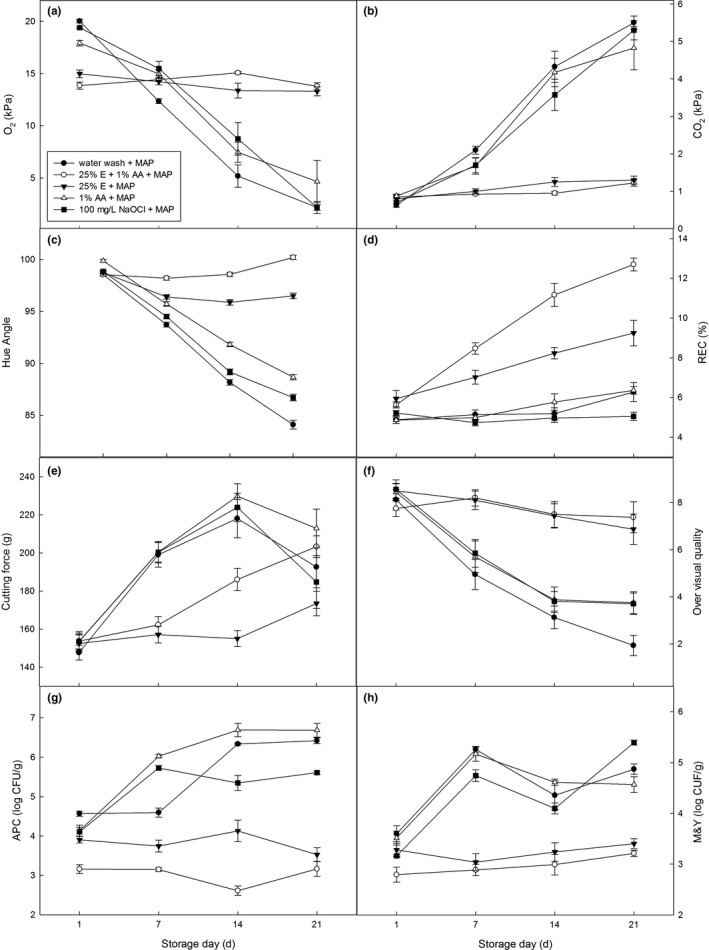
Effect of different dip treatments on the changes in O_2_ (a) and CO_2_ (b) partial pressures within packages, color changes in *hue angles* (c), tissue relative electrical conductivity (d), hardness (e), overall visual quality (f), aerobic plant count (g), and mold & yeast (h) populations of packaged fresh‐cut Chinese yam slices stored at 4°C. Data represent the means ± *SE*. (*n* = 4)

Figure 1a and b show the changes in O_2_ and CO_2_ partial pressures of fresh‐cut Chinese yam slices stored at 4°C for 21 day. The O_2_ and CO_2_ partial pressures for three no ethanol treatments and two treatments containing ethanol were clearly divided into two groups. The no ethanol treatment groups had a sharp O_2_ decline and CO_2_ increase during storage, but the ethanol treatment groups had gentle changes (between 8.23‐12.13 kPa and 1.90‐2.80 kPa, respectively). There were no significant differences in O_2_ and CO_2_ partial pressures between ethanol treatment groups (*p *>* *.01), with or without AA added, but they had significantly higher O_2_ and lower CO_2_ than the three no ethanol treatment groups (*p *<* *.01). The CO_2_ partial pressures for the three no ethanol treatment groups were not significantly different during storage (*p *>* *.01), and O_2_ pressures for 1% AA and NaOCl treatments also were not significantly different (*p *>* *.01) but were significantly higher than water treatment (*p *<* *.05). Headspace gas composition is an indication of the product's respiration rate (Kou et al., 2014). Similar to other studies (Gao et al., 2017; Hu et al., 2010; Wang et al., 2014), ethanol treatment produced a significantly depressed respiration rate in many fresh‐cut vegetables and fruits. Some researchers also found that ethanol could have a slight stimulatory effect on respiration in lettuce (Yan et al., 2015) and sugar cane (Homaida et al., 2017), but it did not influence respiration in our research.

Among the chromatic parameters evaluated, the tonality of the pulp, represented by the hue angle, represents the most suitable indicator of the general appearance of fresh‐cut products as perceived by consumers (Denoya, Vaudagna, & Polenta, 2015). For fresh‐cut Chinese yam slices, a smaller hue angle indicates tissue color change from pale to red/brown (Plotto et al., 2006). In our research, hue angle values for all treatments were significantly different during storage (*p *<* *.01) (Figure 1c). The hue angle for the 25% E + 1% AA treatment did not change significantly before day 21 (*p *>* *.01), which also had the least browning, and the hue angle was significantly higher than for the 25% E treatment (*p *<* *.01), but the 25% E treatment did not change significantly after day 1 (*p *>* *.01). The 1% AA had the third largest change in hue angle, followed by the NaOCl and water wash control. Similar to fresh‐cut lotus root slices (Gao et al., 2017), samples treated with ethanol maintained desirable gas composition and pale color during the storage period, but for samples treated without ethanol, gas composition and color changed greatly (Figure 1a, b and c). Additionally, ethanol and combination treatments had a significantly positive effect on maintaining the color of the fresh‐cut Chinese yam slices, lotus root (Gao et al., 2017), apples (Yan et al., 2017), and sugar cane (Homaida et al., 2017).

Samples treated with 25% E + 1% AA had the highest REC values (12.69%), followed by the 25% E treatment (9.24%) (Figure 1d). The REC values for the three no ethanol treatments had gentle changes (from 4.73% to 6.27%), and there was no significant difference between them during storage (*p *>* *.05). High ethanol in vegetable or fruit tissue may cause accumulation of ethyl esters, which degrade the plasma membrane, increasing ion leakage (Bai, Plotto, Spotts, & Rattanapanone, 2011; Suzuki, Kimura, Takahashi, & Terai, 2005). However, different from fresh‐cut sugar cane and lotus root slices, ethanol and a combination of AA and ethanol did not decrease the REC (Homaida et al., 2017), and added AA in this research also did not reduce the severity of the tissue damage caused by the ethanol (Gao et al., 2017).

To avoid the fiber influencing the texture data, we used an A/MORS blade (9 mm wide and 0.5 mm thickness, with sharp cut edge) to cut the cross‐sections of Chinese yam slices. The texture data for fresh‐cut Chinese yam slices were also divided into two groups. The two treatments containing ethanol showed a trend of gently increased cutting force over time, and the no ethanol treatments sharply increased in cutting force before day 14 but quickly decreased on day 21 (Figure 1e). Cutting forces for the three no ethanol treatment groups were not significantly different during storage (*p *>* *.01) and were higher than the 25% E + 1% AA treatment group before day 14. The 25% E treatment group had the lowest cutting force (*p *<* *.01). The increase in cutting force for fresh‐cut Chinese yam slices may relate to tissue water loss and/or lignifications of Chinese yam slices, and the sharp decrease may have to do with quality deterioration during long storage times. Similar to previous research (Gao et al., 2017; Yan et al., 2017), ascorbic acid combined with ethanol improved cutting force compared to ethanol‐only treatment.

Figure 1f shows the sensory evaluation scores for overall visual quality of the fresh‐cut Chinese yam slices for 21 days of storage. The scores for the two ethanol treatment groups were not significantly different (*p *>* *.01), but they were significantly higher than the three no ethanol treatment groups during storage (*p *<* *.01), and above 6.0 on day 21, while other treatment groups were not salable. Samples treated with 1% AA and NaOCl were not significantly different during storage (*p *>* *.01), but they were higher than the water wash control (*p *<* *.01), with obvious browning and under the salability value after day 7. An alcohol off‐odor was also detected upon opening samples treated with 25% E + 1% AA and 25% E (Bai, 2004; Bai et al., 2011; Gao et al., 2017; Plotto et al., 2006). However, after opening the package, the alcohol smell was quickly lost.

Figure 1g and h show the microbial counts for the fresh‐cut Chinese yam slices after 21 days of storage. The two treatments containing ethanol maintained significantly lower levels of aerobic plate count population (APC) and mold and yeast (M&Y) than the three no ethanol treatments throughout storage. Except the APC for the 25% E + 1% AA treatment on day 14, both APC and M&Y for the two treatments containing ethanol were not significantly different during storage (*p *>* *.01), and the 25% E + 1% AA treatment was significantly lower than the 25% E treatment (*p *<* *.01). The APC and M&Y for 25% E + 1% AA treatment both remained below 3.2 log until after 21 days. However, APC counts for 1% AA and NaOCl treatments increased rapidly during the first week, reaching more than 5.7 log by day 7, and the water wash control increased rapidly during the second week, reaching more than 6.3 log bay day 14. Samples treated with 1% AA only had the highest APC counts during storage (*p *<* *.01). The M&Y counts for no ethanol treatments sharply increased during the first 7 days and then fluctuated within the range of 4.1‐5.4 log until the end of storage, and these three treatments were not significantly different from each other during storage (*p *>* *.01). Similar to the results reported by other researchers, ethanol treatment inhibited microbial growth and limited decay (Bai, 2004; Bai et al., 2011; Homaida et al., 2017; Janisiewicz & Conway, 2010; Lurie et al., 2006; Plotto et al., 2006; Yan et al., 2017), and a combination of ethanol and ascorbic acid significantly improved inhibition of microbial growth compared to ethanol alone (Gao et al., 2017).

### 3.2 Assess efficacy of packing methods

The results for wash treatments (3.1) indicated that 25% E + 1% AA treatment was effective for inhibiting browning and maintaining the quality of fresh‐cut Chinese yam. Vacuum packaging has proved especially suitable for products where oxygen can provoke chemical or biochemical alterations. Therefore, fresh‐cut vegetables and fruits can potentially benefit from this technology, considering that oxygen is one of the substrates of the browning reactions (Denoya et al., 2015). To find a more effective method, VP combining with ethanol and AA dip was tested to investigate their influence on color, REC, texture, and sensory and microbial growth.

**Figure 2:**
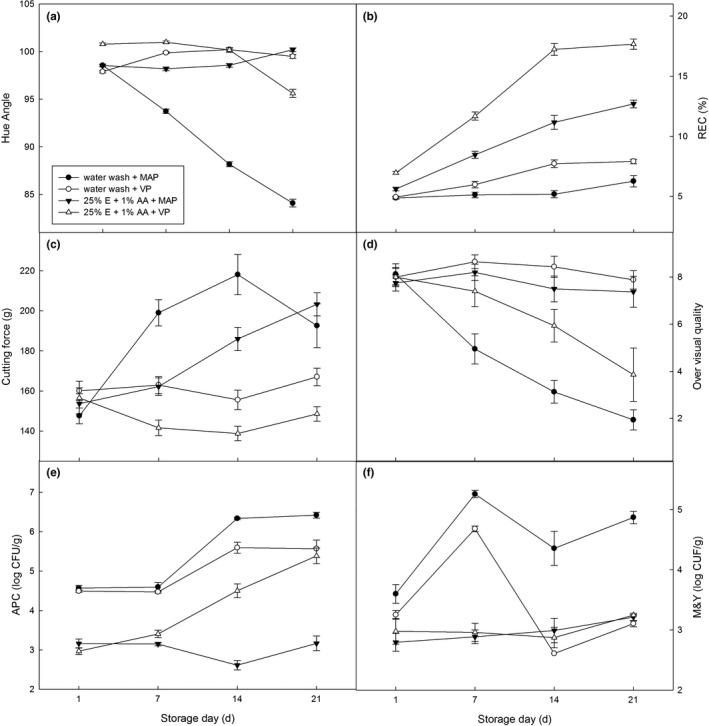
Effect of packing methods on the changes in *hue angles* (a), tissue relative electrical conductivity (b), hardness (c), overall visual quality (d), aerobic plant count (e), and mold & yeast (f) populations of packaged fresh‐cut Chinese yam slices stored at 4°C. Data represent the means ± *SE*. (*n* = 4)

The color measurements for fresh‐cut Chinese yam slices stored at 4°C are shown in Figure 2a. Hue angle values for the water wash + MAP treatment declined significantly with prolonged storage time (*p *<* *.01), but the water wash + VP treatment fluctuated within a small range until the end of storage. Samples treated with ethanol dip with or without VP both had no significant changes before 14 days storage (*p *>* *.01), but the VP treatment groups were significantly higher than MAP treatment groups during storage (*p *<* *.01). Hue angle for the two VP treatments with water wash or ethanol dip was also not significantly different during storage (*p *>* *.01). Color data indicated that VP treatments were more effective at inhibiting browning and reddening than the ethanol dip. It may have resulted in limiting the oxygen concentration and inhibiting polyphenol oxidase (PPO) activity by the vacuum (Denoya et al., 2015).

Figure 2b shows that the REC of all treatment groups increased over storage time, 25% E + 1% AA + VP treatment had the greatest significant increase, 25% E + 1% AA + MAP treatment was second, and water wash + MAP treatment had the lowest REC (*p *<* *.01). The REC for VP treatments was higher than MAP treatments. Experiments with wash treatments have indicated that ethanol treatment may cause cell membrane degradation and increased the REC. This section indicated that VP treatment also may lead to tissue damage for fresh‐cut Chinese yam slices during cold storage, which may be induced by fermentative metabolism and can cause the total alteration of the products (Denoya et al., 2015).

Figure 2c shows the cutting force for the fresh‐cut Chinese yam slices during storage. The two VP treatment groups had little change in cutting force during storage, had lower values than the MAP treatment groups, and the ethanol dip with VP treatment had the lowest cutting force (*p *<* *.01). Cutting force values for water wash + MAP treatment increased sharply before 14 days and dropped quickly on day 21, but the 25% E + 1% AA + MAP treatment group progressively increased quickly after 7 days. The increase in cutting force for MAP treatment groups may be related to tissue moisture loss, but vacuum packing prevented this loss and retained texture characteristics. However, based on the REC data (Figure 2b), ethanol dip and VP treatment may both have resulted in membrane degradation, as they resulted in the lowest cutting force when they were combined.

Figure 2d shows a decrease in overall visual quality for all treatments over the storage period, and the water wash + VP treatment had the highest scores, second to the 25% E + 1% AA + MAP treatment groups that were significantly higher than the scores for other treatment groups (*p *<* *.01) and above 7.0 until end of storage. Samples in the 25% E + 1% AA + VP group lost salability for visual quality after 14 days, and the water wash + MAP group dropped quickly after day 1. Based on the color data (Figure 2a), the hue angles for VP treatment groups were better than others, but there was a severe off‐flavor (data not shown) when we opened the package of 25% E + 1% AA + VP treatments after a long storage time, along with a soft texture, viscous state, and decay, which may be related to accumulation of ethanol and acetaldehyde (Denoya et al., 2015). Samples treated with water wash + VP with a bright white color (Figure 2a) had a slight odor after a long storage time, and texture data (Figure 2c) were better than 25% E + 1% AA + VP treatments. Therefore, a better sensory evaluation score was observed.

Figure 2e and f show the microbial counts for the fresh‐cut Chinese yam slices after 21 days of storage. Treatments containing ethanol maintained significantly lower levels of microbes than treatments without ethanol throughout storage. Samples treated with 25% E + 1% AA + MAP had no significant changes for APC and M&Y counts during storage (*p *<* *.01). The APC counts of 25% E + 1% AA + VP treatment grew rapidly from 14–21 days and reached 5.4 log by day 21. The APC counts of the two water wash treatment groups had no significant changes before 7 days (*p *>* *.01), but sharply increased by day 14, and MAP treatment produced higher microbial counts. The M&Y counts for the two treatments containing ethanol were not significantly different during storage, and both of them did not change significantly (*p *>* *.01). Samples treated with water wash + VP had lower M&Y counts than MAP groups (*p *<* *.01), and they suddenly dropped to lowest levels (2.6‐3.1 log until) from 14–21 days. The results of this section showed that VP with water wash treatment was more effective at inhibiting microbial growth than water wash + MAP treatment. However, VP combined with ethanol dip treatment accelerated APC reproduction in the lag period, which may have been caused by tissue membrane degradation for vacuum packing and fermentative metabolism (Beltrán, Selma, Tudela, & Gil, 2005; Denoya et al., 2015). The inhibition of M&Y growth by 25% E + 1% AA + VP treatment was related to bactericidal action of ethanol, and the sharp decrease in M&Y counts for the water wash + VP treatment after 7 days may have been caused by lower oxygen concentration in the package and competition between bacteria. It follows that the combination of vacuum packaging and ethanol–ascorbic acid washing was necessary to achieve efficient antibrowning and antimicrobial control for fresh‐cut Chinese yam slices stored cold for 7 days but was not suitable for longer storage periods.

## 4 CONCLUSION

The majority of measured data for O_2_ and CO_2_ gas, color, REC, overall visual quality, and microbial populations divided into two groups contained little or no ethanol. The groups that contained ethanol maintained stable values, and no ethanol groups sharply changed after 7 days. A combination of ethanol and ascorbic acid had greater antibrowning and antimicrobial effects on fresh‐cut Chinese yam slices during cold storage than ethanol or ascorbic acid alone, but not for relative electrical conductivity.

Vacuum packaging was better than modified atmosphere packaging for antibrowning of fresh‐cut Chinese yam slices at 4°C but was not effective at controlling the increase in relative electrical conductivity and microbial growth at lag storage. Samples treated with vacuum packaging and 25% ethanol with 1% ascorbic acid dip had preserved sensory qualities of fresh‐cut Chinese yam slices up to 7 days at 4°C, and the same washing treatment replaced by modified atmosphere packaging can both inhibit browning and maintain quality up to 14‐21 days.

## CONFLICT OF INTEREST

None declared.
